# Nutrition and Physical Activity in Aging, Obesity, and Cancer

**DOI:** 10.1111/j.1749-6632.2012.06737.x

**Published:** 2012-10-10

**Authors:** Stephen D Hursting, Sarah M Dunlap

**Affiliations:** 1 Department of Nutritional Sciences, University of TexasAustin, Texas; 2 Department of Molecular Carcinogenesis, University of Texas-MD Anderson Cancer CenterSmithville, Texas

**Keywords:** obesity, cancer, growth factors, inflammation, microenvironment

## Abstract

Obesity is an established risk and progression factor for many cancers. In the United States more than one-third of adults, and nearly one in five children, are currently obese. Thus, a better understanding of the mechanistic links between obesity and cancer is urgently needed to identify intervention targets and strategies to offset the procancer effects of obesity. This review synthesizes the evidence on key biological mechanisms underlying the obesity–cancer association, with particular emphasis on obesity-associated enhancements in growth factor signaling, inflammation, and perturbations in the tumor microenvironment. These interrelated pathways and processes represent mechanistic targets for disrupting the obesity–cancer link.

## Introduction

Obesity is often defined by a body mass index (BMI) of ≥30 kg/m^2^, and its prevalence has increased dramatically over the past 30 years in the United States and many parts of the world. Approximately 35% of adults and 20% of children in the United States are currently obese.^1^ The majority of obese individuals meet the criteria for the metabolic syndrome, a state of metabolic dysregulation characterized by increased waist circumference, insulin resistance, hyperglycemia, hypertension, and hypertriglyceridemia.^2^ Increased circulating levels of insulin, bioavailable insulin-like growth factor (IGF)-1, leptin, inflammatory factors, and vascular integrity-related factors such as vascular endothelial growth factor (VEGF) and plasminogen activator inhibitor (PAI)-1, are typically observed in obese individuals.^3^^,^^4^ In addition, changes in the tumor (and adjacent normal tissue) microenvironment associated with increased cancer susceptibility or enhanced tumor progression, including factors associated with the epithelial-to-mesenchymal transition, have also been characterized.^5^^,^^6^ At least in part through these interacting pathways and processes, obesity increases the risk and/or worsens the outcome of several chronic diseases,^3^^,^^5^ including cardiovascular disease, type II diabetes, and the focus of this review, cancer.

Obesity prevention or reversal is a major part of several evidence-based cancer prevention guidelines.^7^ An estimated 15–30% of cancer deaths in the U.S. population are attributed to the high prevalence of overweight and obese Americans,^8^ with the evidence strongest for endometrial cancer, postmenopausal breast cancer, colon cancer, renal cell carcinoma of the kidney, liver, gallbladder, esophageal, and pancreatic cancer, with mounting evidence for cervical, ovarian, prostate (prognosis, but not risk), and stomach cancer.^7^ This review focuses on possible mechanisms underlying the associations between obesity and cancer, with emphasis on obesity-associated enhancements in growth signaling, inflammatory processes, and vascular perturbations, and microenvironmental disruptions, all linked with cancer susceptibility and poor prognosis.

## Growth signal dysregulation

### Insulin and IGF-1

Hyperinsulinemia and/or hyperglycemia are hallmarks of the obese state and are associated with insulin resistance, aberrant glucose metabolism, chronic inflammation, and the production of other metabolic hormones such as IGF-1, leptin, and adiponectin.^9^ Insulin is a peptide hormone produced by the beta cells of the pancreas and released in response to increased blood glucose, IGF-1 is a peptide growth factor that shares ∼50% sequence homology with insulin and is produced primarily by the liver following stimulation by growth hormone. However, hyperinsulinemia and hyperglycemia can also lead to increased hepatic IGF-1 production independent of growth hormone. IGF-1 regulates development and growth of many tissues, particularly during the prenatal period. IGF-1 in circulation is typically bound to IGF binding proteins (IGFBPs) that regulate the amount of free IGF-1 bioavailable to bind to the IGF-1 receptor (IGF-1R) and elicit growth or survival signaling.^10^ With obesity, the amount of bioavailable IGF-1 increases, possibly via hyperglycemia-induced suppression of IGFBP synthesis and/or hyperinsulinemia-induced promotion of hepatic growth hormone receptor expression and IGF-1 synthesis.^10^^,^^11^ Elevated circulating IGF-1 is an established risk factor for many cancer types.^11^

### Insulin receptor and IGF-1R signaling pathways

The phosphatidylinositol-3 kinase (PI3K)/Akt pathway downstream of the insulin receptor and IGF-1R comprise a signaling network that regulates (and integrates) cellular growth, survival, and metabolism. Cantley *et al.*^12^ established that this signaling cascade is one of the most commonly altered pathways in human epithelial tumors. Engagement of the PI3K/Akt pathway allows both intracellular and extracellular cues about substrate availability, growth factor supply and other factors that impact cell survival, growth, proliferation, and metabolism. Activation of receptor tyrosine kinases, such as the insulin receptor or IGF-1R, stimulates PI3K to produce lipid messengers that facilitate activation of the Akt cascade. Akt regulates the mammalian target of rapamycin (mTOR),^13^ which regulates cell growth, cell proliferation, and survival through downstream mediators. mTOR activation is inhibited by increased AMP-activated kinase (AMPK) under low nutrient conditions.^14^^,^^15^ Increased activation of mTOR is common in tumors and many normal tissues from obese and/or diabetic mice, and specific mTOR inhibitors block the tumor-enhancing effects of obesity in mouse models.^15^^–^^17^

### Leptin, adiponectin, and their ratio

Leptin is a peptide hormone produced by adipocytes, which is positively correlated with adipose stores and nutritional status and functions as an energy sensor to signal the brain to reduce appetite. In the obese state, adipose tissue overproduces leptin, and the brain no longer responds to the signal. Insulin, glucocorticoids, tumor necrosis factor-alpha (TNF-α), and estrogens all stimulate leptin release.^18^ Leptin has direct effects on peripheral tissues, indirect effects on hypothalamic pathways, and modulates immune function, cytokine production, angiogenesis, carcinogenesis, and other biological processes.^18^ The leptin receptor has similar homology to class I cytokines that signal through the janus kinase and signal transducer activator of transcription (JAK/STAT) pathway that is often dysregulated in cancer.^19^

Adiponectin is a hormone mainly secreted from visceral adipose tissue. Levels of adiponectin, in contrast with leptin, negatively correlate with adiposity. Adiponectin functions to counter the metabolic program associated with obesity and hyperleptinemia by modulating glucose metabolism, increasing fatty acid oxidation and insulin sensitivity, and decreasing production of inflammatory cytokines.^20^ The possible mechanisms through which adiponectin exerts anticancer effects may include increasing insulin sensitivity, and decreasing insulin/IGF-1 and mTOR signaling via activation of AMPK. Adiponectin also reduces proinflammatory cytokine expression via inhibition of the nuclear factor kappa-light-chain-enhancer of activated B cells (NF-κB).^20^^–^^22^

*In vitro*, animal and epidemiologic evidence linking leptin^21^^,^^23^^–^^26^ or adiponectin^21^^,^^27^^–^^31^ individually to cancer risk is mixed. Associations between the adiponectin-to-leptin ratio and metabolic syndrome and several cancers^32^^–^^34^ have also been reported, but there are insufficient data thus far to assess the strength of this relationship.

## Chronic inflammation

### Cytokines and crosstalk between adipocytes, macrophages, and epithelial cells

Obesity and metabolic syndrome are associated with a low-grade, chronic (smoldering) state of inflammation characterized by increased circulating free fatty acids and chemoattraction of immune cells (such as macrophages that also produce inflammatory mediators) into the local milieu.^35^^–^^37^ These effects are further amplified by the release of inflammatory cytokines such as interleukin (IL)-1β, IL-6, TNF-α, and monocyte chemoattractant protein (MCP)-1. Adipocytes can enlarge past the point of effective oxygen diffusion, which results in hypoxia and eventually necrosis. Free fatty acids escape the engorged/necrotic adipocytes and deposit in other tissues, which in turn promotes insulin resistance and diabetes (through downregulation of insulin receptors and glucose transporters), hypertension, and fatty liver disease and also activates signaling molecules involved in epithelial carcinogenesis, such as NF-κB.^35^^–^^37^

NF-κB is a transcription factor that is activated in response to bacterial and viral stimuli, growth factors, and inflammatory molecules (e.g., TNF-α, IL-6, and IL-1β), and is responsible for inducing gene expression associated with cell proliferation, apoptosis, inflammation, metastasis, and angiogenesis. Activation of NF-κB is a common characteristic of many tumors and is associated with insulin resistance and elevated circulating levels of leptin, insulin, and/or IGF-1.^37^^–^^40^

## Inflammation and cancer

The link between chronic inflammation and cancer development was first noticed more than 100 years ago by Rudolph Virchow, who observed an abundance of leukocytes in neoplastic tissue.^41^ Now, several tissue-specific inflammatory lesions are established as neoplastic precursors for invasive cancer, including gastritis for gastric cancer, inflammatory bowel disease for colon cancer, and pancreatitis for pancreatic cancer.^42^^,^^43^

Tumor microenvironments are composed of multiple cell types including epithelial cells, fibroblasts, mast cells, and cells of the innate and adaptive immune system.^43^^,^^44^ As discussed previously, macrophages, which are activated in the obese state, infiltrate tumors, and amplify the inflammatory tumor microenvironment through production of cytokines, prostaglandins, and angiogenic factors.^37^^,^^44^ Another important cancer-related inflammatory mediator is cyclooxygenase (COX)-2, an enzyme that is upregulated in most tumors and catalyzes the synthesis of the potent inflammatory lipid metabolite, prostaglandin E_2_. COX-2 overexpression is an indicator of poor prognosis in multiple cancer types.^45^

## Vascular integrity-related factors

### PAI-1

PAI-1 is a serine protease inhibitor produced by endothelial cells, stromal cells, and adipocytes in visceral white adipose tissue.^46^ Increased circulating PAI-1 levels, frequently found in obese subjects, are associated with increased risk of atherogenesis and cardiovascular disease, diabetes, and several cancers.^4^^,^^46^ PAI-1, through its inhibition of urokinase-type and tissue-type plasminogen activators, regulates fibrinolysis and integrity of the extracellular matrix. PAI-1 is also involved in angiogenesis and thus may contribute to obesity-driven tumor cell growth, invasion, and metastasis.^4^

### VEGF

VEGF, a heparin-binding glycoprotein produced by adipocytes and tumor cells, has angiogenic, mitogenic, and vascular permeability-enhancing activities specific for endothelial cells.^47^ Circulating levels of VEGF are increased in obese, relative to lean, humans and animals, and increased tumoral expression of VEGF is associated with poor prognosis in several obesity-related cancers.^48^ The need for nutrients and oxygen triggers tumor cells to produce VEGF, which leads to the formation of new blood vessels to nourish the rapidly growing tumor and facilitate the metastatic spread of tumors cells.^49^

Adipocytes communicate with endothelial cells by producing a variety of proangiogenic and vascular permeability-enhancing factors. These include VEGF, IGF-1, PAI-1, leptin, hepatocyte growth factor, and fibroblast growth factor-2.^49^ In the obese, nontumor setting, these factors stimulate neovascularization in support of the expanding fat mass. These adipose-derived factors may also contribute to obesity-associated enhancement of tumor angiogenesis. However, the relative contributions of tumor-derived, versus adipocyte-derived, proangiogenic factors in tumor development, progression, and metastasis remain unclear.

## Tumor microenvironment

### Epithelial-to-mesenchymal transition

Mouse model studies of cancer often involve xenotransplantation of human tumor cell lines into immunodeficient mice. However, xenograft models are extremely limited due to their lack of normal tumor microenvironment. Immunodeficient mice have aberrant mammary gland development, lack normal immune/inflammatory responses, and resist developing diet-induced obesity (DIO) and calorie restriction (CR) phenotypes, which prevents elucidation of the link between energy balance and cancer development/progression in this system. One approach to overcoming these limitations is the development of syngeneic transplant models in immunologically intact animals for studying the energy balance–cancer link. For example, a recent publication from our laboratory^6^ describes the development and characterization of a transplant model of claudin-low and basal-like mammary cancers, which overcomes many existing limitations of xenograft models by using (1) cells derived from a spontaneous MMTV-Wnt-1 mouse mammary tumor that, like basal-like breast cancers in women, are responsive to obesity-related signals,^50^^,^^51^ and (2) a wild-type, syngeneic host with normal immune function, mammary gland development, and metabolic responses to energy balance modulation.

Our findings in this model^6^ indicate a mechanistic link between energy balance, the epithelial-to-mesenchymal transition (EMT), and tumor initiating cells (TICs) in breast cancer progression. We speculate that DIO prepares fertile soil (tumor microenvironment), including changes in EMT, intratumoral adipocytes, local and systemic hormones, growth factors, and cytokines, for enhanced tumor progression, and that determinants of growth in this fertile soil include the plant variety (intrinsic breast cancer subtype) and/or the seed density (extent of TIC enrichment). In contrast, CR may discourage tumor progression by acting on the soil antithetically to DIO, including promoting epithelial differentiation, discouraging EMT, preventing intratumoral adipocytes infiltration, and decreasing systemic hormones, growth factors, and cytokines. Future studies are warranted to determine whether the tumor-enhancing effects of DIO depend on the extent of TIC-enrichment in different subtypes of breast cancer, as well as other epithelial cancers, and whether CR targets different (and perhaps TIC-independent) pathways than DIO to impact breast cancer progression.

Components of EMT may thus represent novel targets for preventing and/or controlling cancer, and novel biomarkers of response to energy balance modulation or other interventions, particularly in obese women. Although IGF-1, adipocytokines, inflammation, and other obesity-related factors, as well as many cancers, have been individually associated with upregulation of TGF-β and other EMT pathway components,^52^^–^^56^ their combined interactions are poorly characterized. It is highly plausible that energy-responsive growth signaling pathways regulate tumor cell differentiation and TIC proliferation through upregulation of obesity-associated serum hormones and cytokines, including those already described in this review.

## Conclusion

**Figure 1:**
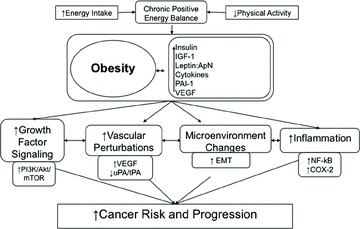
Obesity, energy balance, and cancer: a mechanistic overview. An arrow preceding text denotes a directional effect (e.g., activity or concentration). IGF-1, insulin-like growth factor-1; ApN, adiponectin; PAI-1, plasminogen activator inhibitor-1; tPA, tissue-type plasminogen activator; uPA, urokinase-type plasminogen activator; VEGF, vascular endothelial growth factor; PI3K, phosphoinositide 3-kinase; COX-2, cyclooxygenase-2.

Multiple hormones, growth factors, cytokines, and other mediators associated with the metabolic perturbations of the obese state enable crosstalk between macrophages, adipocytes, endothelial cells, other microenvironmental components, and epithelial cells. As shown in Figure 1, these obesity-associated factors contribute to cancer-related processes (including growth signaling, inflammation, vascular alterations, and several pathways associated with tumor-initiating cell enrichment, invasion, and metastasis). Components of these interrelated processes and pathways represent promising mechanism-based targets for breaking the links between obesity (and its metabolic dysregulation) and cancer.
